# hnRNP‐A1 binds to the IRES of MELOE‐1 antigen to promote MELOE‐1 translation in stressed melanoma cells

**DOI:** 10.1002/1878-0261.13088

**Published:** 2021-09-12

**Authors:** Maud Charpentier, Emilie Dupré, Agnès Fortun, Floriane Briand, Mike Maillasson, Emmanuelle Com, Charles Pineau, Nathalie Labarrière, Catherine Rabu, François Lang

**Affiliations:** ^1^ Inserm LabEx IGO CRCINA Université de Nantes Nantes France; ^2^ Inserm CNRS SFR Santé Inserm UMS 016 CNRS UMS 3556 Université de Nantes Nantes France; ^3^ Inserm EHESP Irset (Institut de recherche en santé, environnement et travail) – UMR‐S 1085 Univ Rennes Rennes France; ^4^ Protim Biosit – UMS 3480 US‐S 018 Univ Rennes Rennes France

**Keywords:** ER stress, IRES, ITAF, long noncoding RNA, melanoma, tumor antigens

## Abstract

The major challenge in antigen‐specific immunotherapy of cancer is to select the most relevant tumor antigens to target. To this aim, understanding their mode of expression by tumor cells is critical. We previously identified a melanoma‐specific antigen, melanoma‐overexpressed antigen 1 (MELOE‐1)—coded for by a long noncoding RNA—whose internal ribosomal entry sequence (IRES)‐dependent translation is restricted to tumor cells. This restricted expression is associated with the presence of a broad‐specific T‐cell repertoire that is involved in tumor immunosurveillance in melanoma patients. In the present work, we explored the translation control of MELOE‐1 and provide evidence that heterogeneous nuclear ribonucleoprotein A1 (hnRNP‐A1) binds to the MELOE‐1 IRES and acts as an IRES trans‐activating factor (ITAF) to promote the translation of MELOE‐1 in melanoma cells. In addition, we showed that endoplasmic reticulum (ER) stress induced by thapsigargin, which promotes hnRNP‐A1 cytoplasmic translocation, enhances MELOE‐1 translation and recognition of melanoma cells by a MELOE‐1‐specific T‐cell clone. These findings suggest that pharmacological stimulation of stress pathways may enhance the efficacy of immunotherapies targeting stress‐induced tumor antigens such as MELOE‐1.

## Graphical abstract

Our study demonstrates that reticular stress in melanoma cells promoted the translocation of the IRES transacting factor hnRPN‐A1 to the cytoplasm. By binding to the IRES site of *meloe* mRNA, hnRPN‐A1 promoted the translation of the melanoma‐specific antigen MELOE‐1 and led to the expression of a new immunogenic HLA/peptide complex at the cell surface. Altogether, our data suggest that pharmacological stimulation of stress pathways may enhance the efficacy of T‐cell based immunotherapies in targeting stress‐induced tumor antigens, such as MELOE‐1 in patients with melanoma.

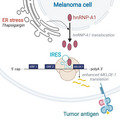

**Abbreviations**
EMCV: encephalomyocarditis virus
ER: endoplasmic reticulum
FCS: fetal calf serum
HLA: human leucocyte antigen
IFNγ: interferon gamma
IRES: internal ribosomal entry sequence
ITAF: IRES trans‐activating factor
SPR: surface plasmon resonance
UPR: unfolded protein response
YFP: yellow fluorescent protein

## 1 Introduction

In cancer immunotherapy, despite the major advance brought by immune checkpoint inhibitors that stimulate broad T‐cell responses [1], it is now clear that further improvements will require an additional specific approach, that is, the activation of T‐cell responses directed against defined tumor antigens. The choice of the target antigens is thus critical, and in recent years, many teams have decided to set aside the differentiation and overexpressed antigens, to focus on antigens exclusively expressed by tumor cells, named ‘neoantigens’. These antigens should represent ideal targets for immunotherapy since normal tissues would then be spared from immunological damage. In fact, vaccination with pools of neoantigens has shown very promising results in melanoma [2, 3]. The neoantigens described so far originated from random genetic alterations such as point mutations in expressed genes [4] or indel mutations generating frameshifts and abnormal peptide translation [5]. The main drawbacks of mutated neoantigens predicted by whole‐genome sequencing and HLA binding algorithms are that (a) a high proportion of them are poorly immunogenic for lack of a corresponding T‐cell repertoire [6, 7], (b) their levels of expression on tumor cells may be too low and not amenable to pharmacological enhancement, and (c) they are often patient specific and thus require expensive personalized treatment.

In the present work, we focused on MELOE‐1, a neoantigen of a new type whose expression is restricted to tumor cells as a result of a combination of lineage‐specific transcription [8] and tumor‐specific IRES‐dependent translation of the polycistronic *meloe* RNA [9]. MELOE‐1 stimulates a broad pre‐existing T‐cell repertoire in melanoma patients against a HLA‐A*0201‐restricted epitope of MELOE‐1 [10] and is involved in immunosurveillance of melanoma [11].

A few years ago, Weingarten‐Gabbay and colleagues reported the discovery of thousands of new sequences whose translation was IRES‐dependent [12] with a high proportion of them located in the 3' untranslated region of human transcripts. Moreover, other polycistronic mRNAs similar to *meloe* RNA, regulated by IRES sequences, were described in mammals [13] and a growing body of evidence suggests that many of so‐called ‘noncoding’ RNAs can also be translated into short polypeptides [14], some of which having *bona fide* physiological roles (reviewed in ref. [15]). We are convinced, as also recently suggested by others [16] that peptides from noncoding regions represent an exploitable source of immunogenic antigens for cancer immunotherapy provided we understand better their mode of translation.

We were thus prompted to explore why and how this IRES translation was initiated in melanoma cells. It is now clear that IRES‐dependent translation is favored in various stress conditions such as DNA damage, amino acid starvation, hypoxia, or endoplasmic reticulum stress [17], conditions that are often met by tumors *in vivo*. A common feature of these stressing stimuli is that they induce an accumulation of misfolded proteins in the endoplasmic reticulum and thus trigger the so‐called unfolded protein response (UPR), a response observed in many different cancer types that may actually promote tumor cell survival and growth. One of the consequences of this UPR is the inhibition of classical protein translation through phosphorylation of EIF2α while favoring IRES‐dependent translation [18, 19] by activating IRES trans‐activating factors (ITAF) [17].

In the present work, we set up to better characterize the structure of MELOE‐1 IRES, to identify for potential ITAFs, and to assess the effect of ER stress on its activation.

## 2 Materials and methods

### 2.1 Cell lines and reagents

Melanoma cell lines (M6, M113, M117, M134, M170) were established from fragments of metastatic tumors and registered in the Biocollection PC‐U892‐NL (CHU Nantes). They were grown in RPMI 1640 containing 10% fetal calf serum (FCS) (Sigma, Lyon, France), 2 nm l‐glutamine, 100 UI·mL^−1^ penicillin, and 0.1 mg·mL^−1^ streptomycin (Gibco, Thermo Fisher Scientific, Illkirch Graffenstaden, France). The T‐cell clone M170.48 recognizing the MELOE‐1_36‐44_ epitope in HLA‐A*0201 and the clone 10C10 recognizing the MART1_26‐35_ epitope in HLA‐A*0201 were grown in RPMI 1640 containing 8% human serum and supplemented with 150 UI of IL‐2. The M117‐YFP cell line was generated by stably transfecting the M117 melanoma cell line with the expression vector pcDNA3 encoding a full length *meloe* cDNA in which MELOE‐1 open‐reading frame (ORF) was replaced by mCitrine YFP (AEM37510.1) (GeneArt, Thermo Fisher Scientific). Following G418 selection, transfected cells were cloned and a stable clone was used in the experiments. Thapsigargin was purchased from Sigma‐Aldrich. Recombinant His‐tagged hnRNP‐A1 protein was purchased from Abcam (ab123212; Paris, France).

### 2.2 Surface plasmon resonance analysis

#### 2.2.1 *In vitro* transcription

MELOE‐1 intercistronic region IR_1215‐1490_ (275 nt IRES region) [20] was cloned into a pBSSK vector under the control of a T7 promoter. It was linearized with NotI and *in vitro* transcribed according to the MegaShort Script Kit (Ambion, Thermo Fisher Scientific) protocol. RNA (0.2 nmol) was biotinylated using the 5’end modification kit and biotin maleimide (Vector Laboratories, Eurobio Scientific, Les Ulis, France) following the manufacturer’s guidelines.

#### 2.2.2 Microrecovery analysis for further MS identification

The biosensor used in this study was a Biacore T200 instrument (GE Healthcare, Limonest, France). Streptavidin Research Grade Sensor Chips (carboxymethyl‐dextran derivatized with streptavidin surface) and HBS‐N (0.01 HEPES, pH 7.4, 0.15 m NaCl) running buffer were also purchased from GE Healthcare and used in all BIA/MS experiments. All surface plasmon resonance (SPR) experiments were performed at a flow rate of 5 µL·min^−1^ at 37 °C. The 275 nt biotinylated RNA sequence upstream of MELOE‐1 ORF was coupled onto the streptavidin surface following the standard biotin‐streptavidin coupling protocol (according to supplier’s procedures) to achieve a residual coupling response of around 1500 RU. Fresh whole cell lysates (10 mm Tris/HCl pH = 7.4, 1.5 mm MgCl2, 1 mm KCl, 0.5 mm DTT, 0.05% NP‐40 supplemented with protease inhibitor) from three melanoma cell lines M117, M134, and M170 were diluted 10‐fold in HBS‐N and injected over the RNA‐coated chip for 5 min. Following the end of sample injection, we proceeded to a microrecovery method to elute bound molecules from the biosensor; that is, we injected a small amount (1 µL) of elution solvent (30 mm NaOH) separated from the running buffer by two air bubbles. Using this sandwich elution, bound molecules were recovered without dispersion. Each lysate sample was run for 40 cycles per rounds using this microrecovery procedure in order to recover enough bound materials for further mass spectrometry identification. Recovered material from 40 round microrecovery procedures eluted in low binding tubes (Protein LoBind Tubes, Eppendorf, Montesson, France) was frozen and subsequently analyzed by nanoliquid chromatography coupled with tandem mass spectrometry (NanoLC‐MS/MS).

#### 2.2.3 SPR binding analysis

The three variants of the 275 nt biotinylated RNA sequence upstream of MELOE‐1 ORF, that is, wild‐type (wt IRES sequence, variant 1, and variant 2) were coupled at about 1500 RU each on three different flow cells of the streptavidin surface following the standard biotin‐streptavidin coupling protocol. Recombinant hnRNP‐A1 protein was diluted in HBS‐EP (0.01 m HEPES, pH 7.4, 0.15 m NaCl, 0.005% (v/v) surfactant P20, 3 mm EDTA) at concentrations ranging from 1.95 to 250 nm and injected over the chip‐bound RNA sequences in a kinetics mode. Flow rate was set up at 30 µL·min^−1^ and association and dissociation allowed for 3 and 10 min, respectively. A 30 mm NaOH solution was injected over the chip for 30 s for regeneration between each cycle. Rmax value (RU), *k*_on_ (M^−1^·s^−1^), *k*_off_ (s^−1^), and *K*d (M) were calculated from kinetic sensorgrams using the Langmuir 1 : 1 model.

### 2.3 NanoLC‐MS/MS analyses

Each SPR recovery sample was subjected to enzymatic digestion. First, proteins were reduced with 1.6 mm DTT in 50 mm ammonium bicarbonate pH 8.5 (15 min at 37 °C) then alkylated with 25 µL of 3.4 mm iodoacetamide in 50 mm ammonium bicarbonate pH 8.5 (15 min at room temperature in the dark). The sample was then digested with 0.05 µg of modified trypsin (Promega, La Farlede, France) at 37 °C overnight. The peptide mixture was finally injected in an Orbitrap instrument (LTQ‐OrbitrapXL, Thermo Scientific) as previously described [21].

The Orbitrap MS data were processed with the mascot distiller v2.6.1.0 software (Matrix Science, London, UK). Peptide and protein identification were then performed using the mascot (mascot server v2.5.01; http://www.matrixscience.com) database search engine and its automatic decoy database search to calculate a false discovery rate (FDR). MS/MS spectra were compared with UniProt KB human proteome database UP000005640 (around 20 000 sequences) and a common contaminant database (247 sequences). Mass tolerance for MS and MS/MS was set at 10 ppm and 0.5 Da. The enzyme selectivity was set to full trypsin with one miscleavage allowed. Protein modifications were fixed carbamidomethylation of cysteines, variable oxidation of methionine. Identification results from Mascot (.dat files) were imported into the proline studio software [22]. This software was then used to validate protein identification with a peptide rank = 1, a FDR of 1% on the score at the peptide spectrum matches level and at least 2 specific peptides.

### 2.4 Immunoprecipitation

10 × 10^6^ melanoma cell lines were UV cross‐linked (254 nm, 150 mJ·cm^−2^, UV crosslinker Vilber BLX‐312, Marne la Vallée, France) and solubilized in 10 mm Tris/HCl pH = 7.4, 1.5 mm MgCl2, 1 mm KCl, 0.5 mm DTT, 0.05% NP‐40 supplemented with protease inhibitor and RNAsin, in a Dounce homogenizer (pestle B, 30 strokes). Cytosolic lysate was recovered by successive centrifugation at 2000 ***g*** (10 min) and 10 000 ***g*** (15 min) and immunoprecipitated with 1 µg of polyclonal anti‐hnRNP‐A1 antibody (rabbit polyclonal PA528385, Thermo Fisher Scientific). Recovered material was denatured (5 min, 95 °C) and was subjected to RT (Superscript III) and nested PCR using the following *meloe*‐specific primers (1^st^ round: fwd 5′‐TTCAGAAGAGAATTCCCCG and rev 5′‐GTTTGCTCCAAAGCATCTAA; 2^nd^ round: fwd 5′‐TTGCAGAACTTGTACAAATC and rev 5′‐GTGGTCAATGCTGATGT).

### 2.5 Bicistronic assays

Bicistronic assays were performed using the Renilla/Firefly expression plasmid (pRF) in which were cloned either wt MELOE‐1 IRES, defined as 275 nucleotides upstream of MELOE‐1 (ORF 1491–1631), or the IRES from EMCV virus used as a positive control [9]. The empty pRF vector was used as negative control. Melanoma (M113) cells were seeded in RPMI‐10% FCS one day prior to transfection to reach 50–70% confluency and were transfected with the different pRF constructs (200 ng per well in 96‐well plate) using 0.4 µL of LTX Lipofectamine (Thermo Fisher Scientific). Cells were lysed 48 h post‐transfection, and luminescence activities (Renilla and Firefly) were measured with a FLUOstar Omega apparatus (BMG LabTech, Champigny sur Marne, France) using the dual reporter assay as instructed (Promega). Results are expressed as the ratio Renilla/Firefly*100. Silencing of hnRNP‐A1 was performed by cotransfecting 50–100 nm of hnRNP‐A1‐specific siRNA (100 nm of sc‐270345, Santa‐Cruz Biotechnology (Heidelberg, Germany) or 50 nm of Hs‐HNRPA1‐1, functionally validated FlexiTube siRNA, Qiagen, Courtaboeuf, France) or a universal siRNA as negative control (sc‐37007, Santa‐Cruz Biotechnology and AllStars Negative control FlexiTube siRNA Qiagen).

### 2.6 RT‐qPCR

Total RNA was extracted from 3 × 10^4^ M113 48 h after siRNA transfection (RNeasy kit, Qiagen). 1 µg of DNAse treated‐RNA was retrotranscribed into cDNA using oligodT primer following manufacturer’s instructions (RevertAid H Minus Reverse Transcriptase). 25 ng of cDNA was used as a template for qPCR analysis (Master Mix SYBR PCR) on a Mx3005P apparatus (Agilent Technologies, Les Ulis, France) using the following oligonucleotides primers (Sigma‐Aldrich): hnRNP‐A1fwd: AACCAAGGTGGCTATGGCG, hnRNP‐A1rev.: TCTGGCTCTCCTCTCCTGC, RPLP0fwd GTGATGTGCAGCTGATCAAGACT, RPLP0rev: GATGACCAGCCCAAAGGAGA. Thermal cycling was consisted of an initial step of 5 min at 95 °C, followed by 40 cycles at 95 °C for 30 s, 30 s at 66 °C, and 72 °C for 30 s and a final elongation of 5 min at 72 °C. Mean threshold cycle (*C*_t_) values from triplicate qPCR were normalized to mean *C*_t_ value of RPLP0. Relative expression of transcripts for each cell line was further normalized to the mean expression of untransfected M113 ($2^{‐\Delta \Delta C_{t}}$method).

### 2.7 Fluorescence analysis

The M117‐YFP cell line was seeded into a 1‐µm slide (8 well) microscopy chamber (IbiDi, CliniSciences, Nanterre, France) plate to reach 50% confluency. The next day, adherent cells were treated for 24 h with thapsigargin at various concentrations. Nuclei were stained with Hoechst 33342 (5 µg·mL^−1^, Life technology), and fluorescence was analyzed with a confocal Nikon A1R (lens 20 × 0.7 Plan Apo). The ratio of YFP‐positive cells to Hoechst 33342‐positive nuclei was calculated using an in‐house algorithm developed by the MicroPiCell imaging facility. On average, over 3000 nuclei were counted on an area of 0.1 cm^2^.

### 2.8 Western blots

M117 melanoma cells were treated or not with thapsigargin (0.5 µm) for 24 h, and cytoplasmic and nuclear lysates were prepared as described [26]. Briefly, buffer 1 (Tris/HCl pH8 20 mm, NaCl 150 mm, EDTA 2 mm, Na3VO4 2 mm, NP‐40 0,1%, glycerol 10%, protease inhibitor cocktail) was used to solubilize cytoplasmic fractions. Nuclear extract was prepared by solubilizing the remaining pellet with high salt buffer 2 (Tris/HCl pH7,9 20 mm, NaCl 420 mm, KCl 10 mm, Na3VO4 2 mm, EDTA 1 mm, glycerol 20%). 5 µg of cytosolic or nuclear extract was loaded on a SDS/PAGE analytical gel. hnRNP‐A1 was detected with polyclonal rabbit anti‐hnRNP‐A1 followed by HRP‐conjugated goat F(ab')2 anti‐mouse/anti‐rabbit IgG (Jackson ImmunoResearch, Suffolk, UK). Anti‐alpha tubulin mAb (sc‐23948, Santa‐Cruz Biotechnology) was used as loading control. Quantification of expression of hnRNP‐A1 was performed with Image Lab software (Bio‐Rad, Marnes La Coquette, France) using cytoplasmic expression in untreated cells as a reference and adjusted with tubulin expression in each sample.

### 2.9 T‐cell clone assay

T‐cell clones M170.48 and 10C10 were stimulated for 5 h in the presence of brefeldin A (10 µg·mL^−1^, Sigma) with the tumor cell lines M113 and M6 (pretreated or not with thapsigargin for 24 h and washed extensively over another 24 h period) at an E : T ratio of 1 : 2. Cells were first stained with a PE‐conjugated anti‐CD8 mAb (BioLegend, clone RPA‐T8), fixed with 4% paraformaldehyde (Electron Microscopy Sciences), permeabilized with saponin 0.1%, stained with an APC‐conjugated anti‐ IFNγ mAb (BioLegend, clone B27) as previously described [11], and analyzed by flow cytometry. The expression of HLA‐A*0201 was assessed by flow cytometry using a PE‐conjugated anti‐HLA‐A*0201‐specific antibody (clone BB7.2, BD, Le pont de Claix, France).

### 2.10 Statistical analysis

Data from bicistronic experiments are expressed as mean ± SD and were tested for statistical significance using repeated‐measure one‐way ANOVA followed by Holm–Sidak’s multiple comparison test. Within each experiment, individual values represent the mean of a quadruplicate measure. Data from T‐cell clone assay were tested for statistical significance using a paired *t*‐test. Statistical tests were performed using graphpad prism software (v7.04, San Diego, CA, USA).

## 3 Results

### 3.1 SPR microrecovery and MS analyses

Our previous study on MELOE‐1 translation [9] reported the presence of an IRES activity located around 250 nt upstream of MELOE‐1 ORF. In the present work, we looked for the ITAF(s) that could bind to this region. To this aim, we *in vitro* transcribed the 275 nt RNA sequence upstream of MELOE‐1 ORF, biotinylated it, and coupled it to a streptavidin BIAcore chip. We then prepared whole cell lysates from three melanoma cell lines M117, M134, and M170, ran them on the RNA‐coated BIAcore chip (40 rounds of 5‐min injections), and then recovered the eluted material for mass spectrometry analysis. An average of 100 proteins were identified in each experiment, and among them, we focused on the hnRNP family since members of this large family of RNA‐binding proteins were recurrently recovered in all experiments and this family is known to contain ITAFs. A summary of our results is presented in Table 1, showing consistent recovery of hnRNP‐A1, hnRNP‐F, and hnRNP‐H. While hnRNP‐F/H is described essentially as splicing regulators [18], hnRNP‐A1 has multiple roles including translation control [23, 24] and we thus looked for potential recognition sites of hnRNP‐A1 on the IRES of MELOE‐1.

**Table 1:** hnRNP proteins detected by MS in lysates from melanoma cell lines. Protein identifications were validated with the following criteria: peptide rank = 1, FDR of 1% on the score at the peptide spectrum matches level and at least 2 specific peptides. For each protein, the number of peptides identified and the protein identification score (sum of the unique peptide score calculated following −10log10(p), where *P* is the absolute probability) in brackets are indicated.

	UniProt Accession number	M117			M134			M170	
		Exp1	Exp2	Exp3	Exp1	Exp2	Exp3	Exp1	Exp2
hnRNP‐A1	P09651	6 (245.68)	2 (163.06)	0	9 (358.98)	3 (210.51)	5 (304.23)	7 (333.56)	2 (120.81)
hnRNP‐A2/B1	P22626	0	3 (111.94)	0	3 (122.9)	2 (120.27)	2 (51.78)	3 (119.29)	2 (72.36)
hnRNP‐D	Q14103	0	0	2 (83.26)	3 (67.1)	0	3 (144.79)	3 (101.93)	0
hnRNP‐F	P52597	4 (229.2)	6 (274.6)	8 (432.01)	5 (216.03)	7 (379.62)	8 (403.06)	0	5 (245.06)
hnRNP‐H1	P31943	4 (208.46)	5 (204.76)	7 (282.96)	0	11 (517.42)	9 (387.8)	4 (173.85)	6 (294.38)
hnRNP‐H2	P55795	0	0	0	4 (190.06)	0	6 (270.86)	0	0
hnRNP‐H3	P31942	0	0	0	0	0	2 (67.01)	0	2 (123.98)
hnRNP‐K	P61978	3 (92.93)	2 (61.09)	0	5 (147.71)	0	0	2 (51.47)	7 (229.99)
hnRNP‐M	P52272	15 (615.37)	0	0	10 (333.32)	0	0	4 (92.27)	0

### 3.2 Binding of hnRNP‐A1 to MELOE‐1 IRES

#### 3.2.1 Surface plasmon resonance analyses

**Fig. 1:**
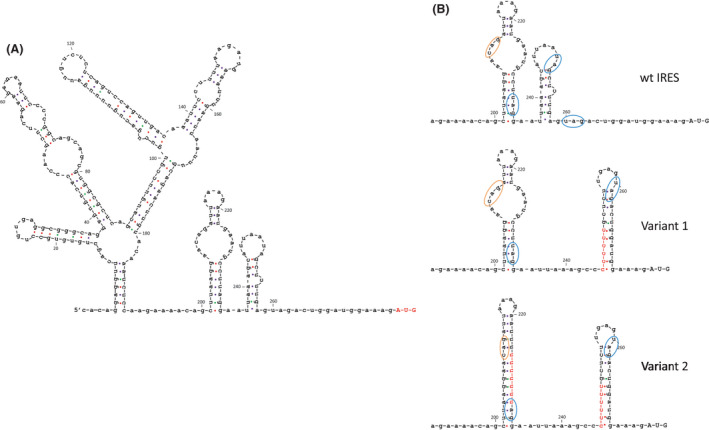
RNA secondary structures upstream of MELOE‐1 ORF, redrawn from predictions by UNAFold (http://www.unafold.org), revealed typical stem‐loop elements and putative hnRNP‐A1 binding sites. (A) Schematic representation of the predicted RNA secondary structure of the 275 nt sequence upstream of MELOE‐1 ORF (AUG initiation codon in red). (B) Focus on the proximal regions of the wt, variant 1, and variant 2 internal ribosomal entry sequence (IRES), highlighting the putative hnRNP‐A1‐binding sites, circled in orange (5′CAG‐3') and blue (5′UAG‐3'). Nucleotide changes in variants 1 and 2, shown in red, are predicted not to change the rest of the IRES sequence.

The secondary structure of the 275 nt sequence upstream of MELOE‐1 ORF was predicted by the online RNA folding form (mfold) application (http://www.unafold.org) and is shown in Fig. 1A. When we looked at the predicted secondary structure, we noticed two stem loops close to the AUG initiation codon that contained multiple potential hnRNP‐A1‐binding sites such as 5'‐UAG‐3' and 5'‐CAG‐3' [19] (Fig. 1B top panel). To assess whether these stem loops were involved in hnRNP‐A1 binding, we designed a variant 1 IRES by replacing the first loop sequence AUUAAUA with CCCCCCC and a variant 2 in which the sequence GAAUGCC was replaced by UUUUUUU to also destroy the second loop as shown in Fig. 1B. The predicted structures of the variant forms suggested that these modifications resulted in the sole destruction of the targeted loops without affecting the rest of the structure.

**Fig. 2:**
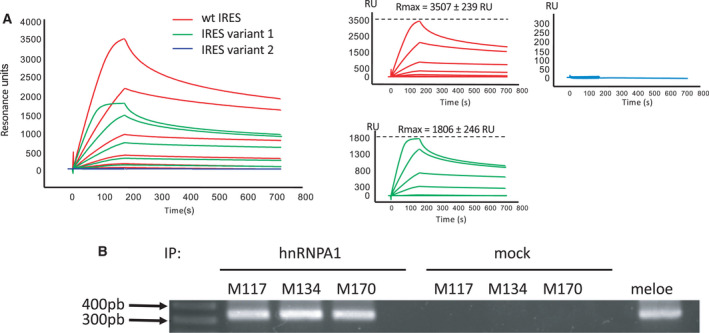
hnRNP‐A1 binds to MELOE‐1 internal ribosomal entry sequence (IRES). (A) Typical surface plasmon resonance (SPR) sensorgram (out of three performed) showing binding of recombinant hnRNP‐A1 (concentrations ranging from 1.95 to 250 nm) to immobilized 275 nt MELOE‐1 wt IRES (red line), variant 1 (green line), and variant 2 (blue line). (B) RT‐PCR detection of *meloe* RNA after immunoprecipitation with anti‐hnRNP‐A1 mAb of UV cross‐linked lysates from M117, M134, and M170 melanoma cell lines. PCR from mock‐immunoprecipitated cell lysates and from plasmid are shown as negative control and positive control, respectively.

We then compared by surface plasmon resonance (BIAcore) the binding of pure recombinant hnRNP‐A1 to our chip‐bound wild‐type IRES, variant 1, and variant 2. Sensorgrams of a typical experiment out of 3 performed are shown in Fig. 2A. Recombinant hnRNP‐A1 bound the wild‐type IRES sequence with good affinity (*k*_on_ = 1.38 × 10^5^ ± 0.64 × 10^5^ M^−1^·s^−1^, *k*_off_ = 1.21 × 10^−3^ ± 0.33 × 10^−3^·s^−1^, *K*d = 9.58 × 10^−9^ ± 2.56 × 10^−9^, *n* = 3) but also bound to the variant 1 IRES with no significant difference in affinity (*k*_on_ = 1.21 × 10^5^ ± 0.33 × 10^5^ M^−1^·s^−1^, *k*_off_ = 1.10 × 10^−3^ ± 0.19 × 10^−3^·s^−1^, *K*d = 9.56 × 10^−9^ ± 2.64 × 10^−9^, *n* = 3). However, the amount of hnRNP‐A1 (Rmax) bound to variant 1 was repeatedly half of the amount bound to the wild‐type IRES (3507 ± 239 for wt IRES vs 1806 ± 246 for variant 1, *n* = 3) and this was not due to differences in the amount of RNA bound to the chip (1.46 × 10^3^ ± 62.29 RU for wt IRES vs 1.49 × 10^3^ ± 91.65 RU for variant 1, *n* = 3). The most likely explanation for these results was that hnRNP‐A1 had two binding sites of similar affinity on wt IRES and only one left on the variant 1 IRES. We thus hypothesized that the neighboring stem loop may provide a second binding site and this was supported by the finding that additional modification of the second loop in variant 2 totally abrogated the binding of hnRNP‐A1 as shown by the sensorgram in Fig. 2A. Altogether, these data demonstrated that hnRNP‐A1 bound MELOE‐1 IRES with good affinity and strongly suggested that hnRNP‐A1‐binding sites were located on the two loops closest to the initiation codon.

#### 3.2.2 Immunoprecipitation

To confirm the binding of hnRNP‐A1 to *meloe* RNA within melanoma cells, we performed immunoprecipitation of cytoplasmic cell lysates with an anti‐hnRNP‐A1 mAb followed by a *meloe*‐specific PCR amplification. As shown in Fig. 2B, in the three melanoma cell lines tested, *meloe* RNA could be amplified thus confirming its physiological association with hnRNP‐1 in these cells.

### 3.3 hnRNP‐A1 promotes the translation of MELOE‐1

**Fig. 3:**
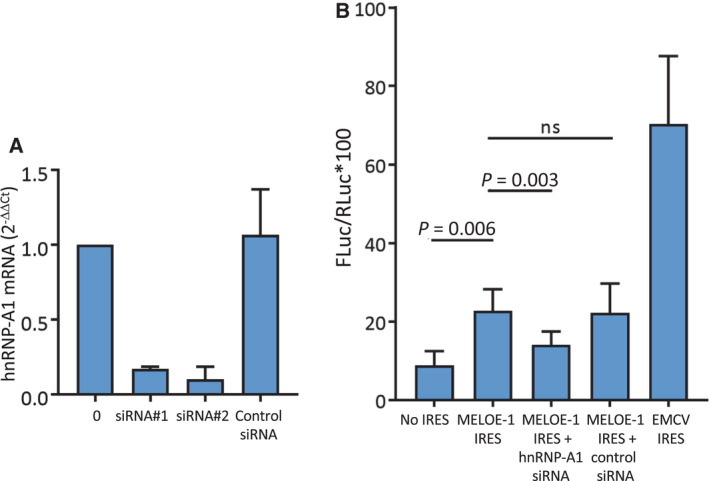
Silencing of hnRNP‐A1 reduces MELOE‐1 internal ribosomal entry sequence (IRES) activity. (A) Efficacy of siRNA‐mediated depletion of hnRNP‐A1 on M113 assessed by RT‐qPCR 48 h postlipofection. (B) FLuc/RLuc ratio (*100) was measured in M113 melanoma cell lysate 48 h post‐transfection with pRF bicistronic vectors in which Renilla luciferase (RLuc) translation is cap‐dependent and Firefly luciferase (FLuc) translation is controlled either by MELOE‐1 IRES, encephalomyocarditis virus (EMCV) IRES, or nothing (no IRES). Where indicated, cells were cotransfected with hnRNP‐A1 siRNA (siRNA#1, 10 µm, Santa‐Cruz Biotechnologies, siRNA#2, 5 µm, Qiagen) or with a universal control siRNA (5–10 µm). Data are expressed as mean ± SD (*n* = 7 independent experiments). *P*‐values were calculated using repeated‐measure one‐way ANOVA followed by Holm–Sidak’s multiple comparison test.

hnRNP‐A1 has been reported to both promote and inhibit the activation of IRES [23], and to formally test the role of hnRNP‐A1 on the translation of MELOE‐1, we used M113 melanoma cells transfected with a Renilla–Firefly bicistronic reporter plasmid, a model that we previously used to assess the IRES‐dependent translation of MELOE‐1 [9]. Translation of Firefly luciferase was under the control of either the 275 bp MELOE‐1 IRES sequence, the EMCV IRES used as positive control, or the empty vector as negative control. In this model, we explored the role of hnRNP‐A1 by assessing the effect of the cotransfection of hnRNP‐A1‐specific siRNAs on MELOE‐1 IRES‐dependent translation in the M113 melanoma cell line. We utilized two different hnRNP‐A1‐specific siRNAs (respectively from Santa‐Cruz Biotechnologies and Qiagen) and checked their silencing abilities on hnRNP‐A1 mRNA expression in the M113 cell line by quantitative PCR. As shown in Fig. 3A, both siRNAs were similarly efficient at suppressing hnRNP‐A1 mRNA expression allowing us to combine the results of the bicistronic assays obtained with the siRNA from Santa‐Cruz Biotechnologies (*n* = 5) and the confirmation experiments with the Qiagen siRNA (*n* = 2). As shown in Fig. 3B, the ratio of luminescence Firefly/Renilla was significantly increased with MELOE‐1 IRES when compared to empty vector (22.7% ± 5.5 vs 8.8% ± 3.5, *P* = 0.006, *n* = 7). Cotransfection with hnRNP‐A1‐specific siRNAs significantly decreased IRES activity (22.7% ± 5.5 vs 14.1% ± 3.3, *P* = 0.003, *n* = 7) while the control siRNA did not significantly affect IRES activity. This strongly suggested that hnRNP‐A1 was indeed an ITAF promoting translation after binding to MELOE‐1 IRES and not an inhibitory ITAF.

### 3.4 ER stress increases hnNRP‐A1 translocation and MELOE‐1 translation in melanoma cells

To simplify the detection of IRES‐dependent translation of MELOE‐1, we stably transfected the melanoma cell line M117 with the full‐length *meloe* cDNA in which the ORF coding for MELOE‐1 was replaced by the sequence coding for the fluorescent protein YFP.

**Fig. 4:**
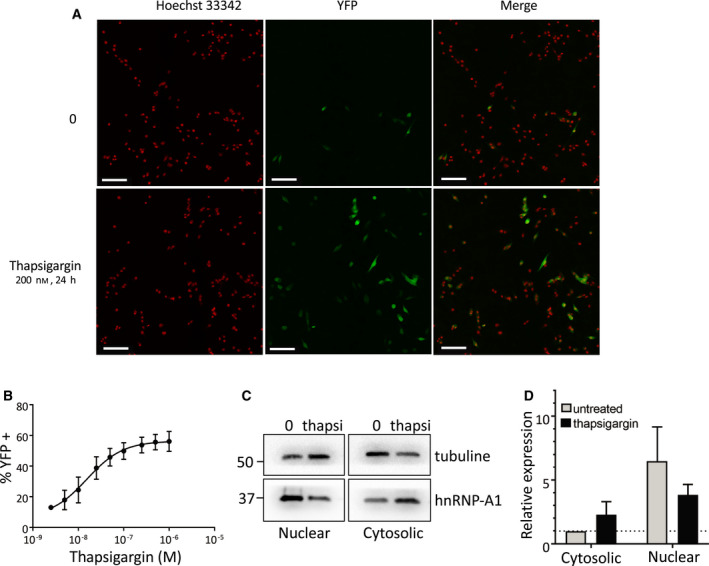
The endoplasmic reticulum (ER) stressor thapsigargin enhances MELOE‐1 expression and hnRNP‐A1 cytosolic translocation. (A) Confocal microscopy detection of yellow fluorescent protein (YFP) expression in the M117‐YFP cell line after a 24‐h thapsigargin treatment (200 nm) or not. M117 was stably transfected with *meloe* cDNA in which MELOE‐1 ORF was replaced by YFP. Nuclei, stained with Hoechst 33342, are shown in red (left panel) and YFP+ cells in green (middle panel). The white bar scale represents 100 µm. (B) Percentages of YFP‐positive cells (expressed as mean ± SD) in response to increasing concentrations of thapsigargin in 6 independent experiments (over 3000 nuclei are counted in each condition). (C) Cellular sublocalization of hnRNP‐A1. M117‐YFP cells were treated with 0.5 µm thapsigargin for 24 h. Cells were harvested, and the nuclear and cytosolic fractions were analyzed by western blot for hnRNP‐A1 and tubulin levels. (D) Quantification of hnRNP‐A1 expression in cytoplasmic and nuclear fractions (*n* = 3 independent experiments, expressed as mean ± SEM). The level of expression of hnRNP‐A1 and tubulin in the cytosolic fraction of untreated cells was used as reference on each blot. Levels of hnRNP‐A1 are expressed relative to the levels of tubulin in each sample (internal control of even total protein loading between samples). Quantification of expression of hnRNP‐A1 was performed with Image Lab software using cytoplasmic expression in untreated cells as a reference and adjusted with tubulin expression in each sample. Considering these adjustments to show relative expression, no statistical test could be performed.

A typical example is shown in Fig. 4A where about 8% of untreated transfected cells spontaneously expressed YFP in culture. Since IRES‐dependent translation is favored in response to ER stress, we decided to use thapsigargin, a calcium pump (SERCA) inhibitor, to induce ER stress in our cells. Indeed, a 24‐h treatment of the cells with 200 nm of thapsigargin, a calcium pump inhibitor used to induce ER stress, resulted in YFP expression in up to 56% of the cells. This stimulating effect of thapsigargin on YFP expression was dose‐dependent as shown in Fig. 4B reaching a plateau around 100 nm, thus demonstrating that ER stress boosted the IRES‐dependent translation of MELOE‐1. In parallel, we confirmed by western blot that thapsigargin induced translocation of hnRNP‐A1 to the cytosol in our cells as was previously described in HeLa cells [24, 25] or HepG2 cells [26]. A typical example is shown in Fig. 4C and the summary of 3 experiments in Fig. 4D expressed as relative expression, using cytoplasmic hnRNP‐A1 in untreated cells as reference after normalization on tubulin expression.

We were thus prompted to assess the effect of ER stress on antigenic recognition of melanoma cells by specific T cells. To this aim, we treated the M113 melanoma cell line with 100 nm of thapsigargin and tested its recognition by a T‐cell clones specific for the MELOE‐1/HLA‐A*0201 epitope (M170.48) and for MART1/HLA‐A*0201 epitope (10C10). The M6 melanoma cell line which is negative for HLA‐A*0201 was used as negative control to ensure that T‐cell activation did not result from the action of remaining thapsigargin released by melanoma cells.

**Fig. 5:**
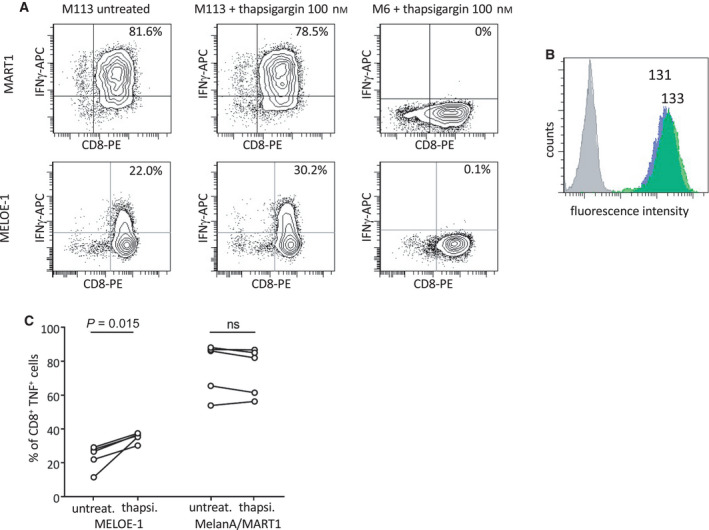
Thapsigargin treatment of the M113 melanoma cell line enhances MELOE‐1 presentation to a specific CD8 T‐cell clone. (A) Interferon gamma (IFN‐γ) production by a Melan‐A/MART1_26‐35 A27L_ (top panel) and MELOE‐1_36‐44_ CD8 T‐cell clone (bottom panel) was measured by intracellular staining after a 5‐h exposure to M113 (HLA‐A*0201 positive) or M6 (HLA‐A*0201 negative) melanoma cell line pretreated or not with thapsigargin (100 nm for 24 h followed by a 24‐h wash‐out period). (B) Thapsigargin treatment of M113 melanoma cell line (as in A) does not affect HLA‐A*0201 expression at the cell surface. Cells were analyzed by flow cytometry after staining with a PE coupled‐anti‐HLA‐A*0201‐specific Ab (gray: isotype control on untreated M113; blue: HLA‐A*0201 on untreated M113; green: HLA‐A*0201 on thapsigargin‐treated M113). Relative fluorescence intensity (RFI) is indicated above each histogram. (C) Compilation of five experiments performed as in (A) showing consistent increase of IFNγ production by MELOE‐1_36‐44_ CD8 T‐cell clone in response to thapsigargin‐treated M113 melanoma cell line (100 nm), while the Melan‐A/MART1_26‐35 A27L_ response is not affected. Data were tested for statistical significance using a paired *t*‐test.

The stress induced by thapsigargin on M113 cells increased their recognition by the MELOE‐1 specific T‐cell clone (as evidenced by IFNγ production) (Fig. 5A, lower panel), with no effect on HLA expression (Fig. 5B). In contrast, thapsigargin had no effect on the recognition of M113 by the Melan‐A/MART1‐specific T‐cell clone. No stimulation was observed with the M6 cell line confirming that the 24‐h wash‐out (see M&M) was efficient to prevent subsequent release of thapsigargin during the recognition assay. Indeed, we observed that thapsigargin can stimulate unspecific T‐cell activation in agreement with a previous report [27]. A summary of 5 independent experiments showed a significant increase in MELOE‐1 recognition (*P* = 0.015) with no significant difference in Melan‐A recognition (Fig. 5C).

These data strongly suggest that in response to ER stress, IRES‐dependent translation of MELOE‐1 and the resulting epitope presentation to T lymphocytes was increased in melanoma cells.

## 4 Discussion

We started our study of the activation of MELOE‐1 IRES by the characterization of the proteins from 3 melanoma cell lines that could bind to the 275 nt long region upstream from the ORF of MELOE‐1, a region previously shown to bear the IRES activity [9]. We used SPR microrecovery at 37 °C to be as close as possible to physiological conditions to allow proper folding of the IRES and interactions with proteins. Nevertheless, we recovered and identified around a hundred different proteins in each experiment: Some of them were devoid of RNA‐binding abilities and obviously contaminants. Considering the sensitivity of mass spectrometry, it was not so surprising to detect unspecific binding of minute amounts of contaminant proteins on the BIAcore chip. Among the proteins with RNA‐binding capacities, the most frequent and recurrent proteins identified were 40S and 60S ribosomal proteins and elongation factors (data not shown) which was consistent with translation initiation in this region. Searching for ITAF, we were more interested in the recurrent detection of hnRNP proteins and especially hnRNP‐A1, recovered in all but one experiment (Table 1) and known to have multiple roles including regulation of IRES [23].

SPR spectroscopy confirmed that recombinant hnRNP‐A1 bound the 275 nt wt IRES with high affinity (Kd around 10 nm). hnRNP‐A1 has two RNA recognition motifs (RRM 1 and RRM 2) with RRM 1 recognizing preferentially the 5'‐UAG‐3' motif and RRM 2 the '5‐CAG‐3' motif [28]. We found these motifs in the two loops closest to the initiation codon on MELOE‐1 275 nt WT IRES. To determine which of these two loops were necessary for hnRNP‐A1 binding, we created variant 1 and variant 2 to destroy one or both loops, respectively, and verified with the online mfold prediction tool that the rest of the IRES structure was conserved. Our results strongly suggested that both loops are involved in hnRNP‐A1 binding but in‐depth analysis with point mutations would be required to determine precisely the binding sites of hnRNPA‐A1 on these loops and whether hnRNP‐A1 dimerizes or not upon binding. However, this investigation was beyond the scope of the present study. In addition, we verified by immunoprecipitation with an anti‐hnRNP‐A1 mAb that hnRNP‐A1 was bound to *meloe* RNA in three melanoma cell lines. Although this observation did not provide information about the role of this association, it confirmed that it is physiologically relevant.

Since hnRNP‐A1 was described both as an activating ITAF [26, 29] and as an inhibitory ITAF [24, 30], we explored the effect of silencing hnRNP‐A1 on MELOE‐1 IRES using a bicistronic Renilla/Firefly reporter assay. We observed a significant decrease in IRES‐dependent translation following cotransfection with two different hnRNP‐A1 siRNAs, thus strongly suggesting that hnRNP‐A1 was an activating ITAF for MELOE‐1 IRES.

Considering the largely reported links between cellular stress, noncanonical translation, and hnRNPA‐A1 [26, 31], we explored the role of ER stress on MELOE‐1 IRES activation. Using the M117‐YFP cell line, we could evaluate the activity of MELOE‐1 IRES by counting of YFP‐positive cells by confocal microscopy. To induce ER stress, we used the specific endoplasmic reticulum Ca2+‐ATPase (SERCA) inhibitor thapsigargin that induces Ca2+ depletion in the reticulum resulting in the unfolded protein response (UPR) and eventually cell death [32]. Thapsigargin dose‐dependently increased IRES‐dependent YFP expression with a maximum effect around 100 nm, a dose consistent with that previously reported to induce ER stress [32]. Thus, ER stress increased very significantly the activation of MELOE‐1 IRES and this was accompanied in those cells by a translocation of hnRNP‐A1 to the cytoplasm, a prerequisite for its action as an ITAF [24].

Finally, we showed that thapsigargin treatment enhanced recognition of the M113 melanoma cell line by a MELOE‐1‐specific T‐cell clone with no significant effect on HLA expression. This increased recognition was antigen‐specific since recognition of Melan‐A/MART1 by the specific clone 10C10 was unchanged. This supports the hypothesis that the effect of thapsigargin was due to a specific increase in MELOE‐1 translation and not an increased expression of T‐cell costimulators on melanoma cells. In fact, the unchanged recognition of Melan‐A was unexpected since we anticipated a decrease in the cap‐dependent translation of the antigen Melan‐A/MART1 in response to thapsigargin leading to a decreased recognition by the clone. A possible explanation is that during the 24‐h wash‐out that was compulsory to prevent thapsigargin release from melanoma cells and nonspecific activation of the clone, melanoma cells recovered and re‐expressed sufficient amounts of MART1/HLA‐A*0201 complexes to stimulate the clone. Still, the main finding was that the induction of reticulum stress in melanoma cells enhanced *in fine* their recognition by T lymphocytes directed against the IRES‐dependent MELOE‐1 antigen. Thapsigargin is too toxic to be a drug candidate but thapsigargin‐based prodrugs such as mipsagargin have been developed and are currently tested in clinical trials in various cancers to exploit their apoptosis‐inducing abilities [33]. It would be interesting to explore whether these compounds could be also used in melanoma patients to enhance the expression of IRES‐dependent antigens such as MELOE‐1 in order to improve immunotherapy.

An alternative would be to stimulate hnRNP‐A1 directly but this is a complex issue since many post‐translation modifications have been reported that affect hnRNP‐A1 activity. For example, asymmetrical methylation of arginine residues within the RGG domain of hnRNP‐A1 by PRMT1 decreases hnRNP‐A1 inhibitory effect on XIAP IRES [34] while symmetrical methylation of the same residues by PRMT5 increases hnRNP‐A1 ability to stimulate IRES‐dependent translation of CCND1, MYC, HIF1a, and ESR1 [35].

In addition, phosphorylation of Ser^199^ by Akt was shown to inhibit hnRNP‐A1 ITAF activity [29]. Since the PI3K/Akt pathway is frequently upregulated in many cancers and a major actor in tumorigenesis, a number of PI3K and Akt inhibitors are currently being evaluated in clinical trials or already approved for clinical use in cancer [36]. We reckon that these drugs through their inhibition of cap‐dependent translation resulting from inhibition of 4EBP phosphorylation by mTORC1 on the one hand and the inhibition of hnRNP‐A1 phosphorylation on the other hand should enhance the expression of MELOE‐1 and similar IRES‐dependent antigens and therefore could provide an additional benefit if combined with immunotherapies targeting those antigens. This remains to be formally investigated. In conclusion, we argue that stress‐induced antigens such as MELOE‐1 whose expression is restricted to tumor cells and depends on ITAF that may be pharmacologically enhanced represent ideal targets for specific cancer immunotherapy.

## 5 Conclusion

In conclusion, our data provide evidence that hnRNP‐A1 behaves as an ITAF binding to and activating the IRES of MELOE‐1, a melanoma‐specific antigen. ER stress induced by thapsigargin promotes hnRNP‐A1 translocation and enhances MELOE‐1 translation and recognition of melanoma cells by T lymphocytes.

## Conflict of interest

The authors declare no conflict of interest.

## Author contributions

According to the CRediT Nomenclature, the following roles are attributed to each author: MC and ED conceptualized and investigated the study, and involved in formal analysis; AF and FB investigated the study; MM and EC investigated the study and involved in formal analysis; CP supervised the study; NL involved in funding acquisition, supervised, and wrote—review and editing; CR conceptualized the study, investigated the study, involved in formal analysis, visualized, and wrote—original draft; FL conceptualized, involved in funding acquisition and project administration, validated, and wrote—original draft (with input from all authors).

### Peer Review

The peer review history for this article is available at https://publons.com/publon/10.1002/1878‐0261.13088.

## Data Availability

Mass spectrometry data are available at Mendeley Data, V1, https://doi.org/10.17632/2m656jrwpn.1.
