# Correction to: hnRNP‐A1 binds to the IRES of MELOE‐1 antigen to promote MELOE‐1 translation in stressed melanoma cells

**DOI:** 10.1002/1878-0261.13635

**Published:** 2024-03-23

In the paper by Charpentier *et al*. [1], additional funding information should have been included within Acknowledgments, which is given below:

This work was supported by the LabEx IGO program (n° ANR‐11‐LABX‐0016) funded by the «Investment into the Future» French Government program, managed by the National Research Agency (ANR).

## References

[1] Charpentier M , Dupré E , Fortun A , Briand F , Maillasson M , Com E , et al. hnRNP‐A1 binds to the IRES of MELOE‐1 antigen to promote MELOE‐1 translation in stressed melanoma cells. Mol Oncol. 2022;16(3):594–606. 10.1002/1878-0261.13088 34418284 PMC8807352

